# Determinants of poor glycemic control among type 2 diabetes mellitus patients at University of Gondar Comprehensive Specialized Hospital, Northwest Ethiopia: Unmatched case-control study

**DOI:** 10.3389/fendo.2023.1087437

**Published:** 2023-02-09

**Authors:** Gebrehiwot Lema Legese, Getahun Asres, Shitaye Alemu, Tesfaye Yesuf, Yeabsira Aklilu Tesfaye, Tsegaw Amare

**Affiliations:** ^1^ Department of Internal Medicine, School of Medicine, College of Medicine and Health Sciences, University of Gondar, Gondar, Ethiopia; ^2^ Department of Health Systems and Policy, Institute of Public Health, College of Medicine and Health Sciences, University of Gondar, Gondar, Ethiopia

**Keywords:** determinants, diabetes mellitus, glycemic control, HbA1C, Ethiopia

## Abstract

**Background:**

Poor glycemic control is one of the most determinant factors for type 2 diabetes-related morbidity and mortality. The proportion of type 2 diabetes mellitus with poor glycemic control remains high. Yet evidences on factors contributing to poor glycemic control remain scarce. The aim of this study is to identify determinants of poor glycemic control among type 2 diabetes mellitus patients at a diabetes mellitus clinic in University of Gondar Comprehensive Specialized Hospital, Northwest Ethiopia.

**Methods:**

A hospital-based case-control study was conducted from June to September 2020. Using convenience sampling techniques, a total of 90 cases and 90 controls with type 2 diabetes were recruited. The data were entered into Epidata version 4.6.0.2 and analyzed by Stata version 14. A multivariable logistic regression analysis was performed to assess the association between independent variables and glycemic control. Both 95% CI and p-value<0.05 were used to determine the level and significance, respectively.

**Results:**

The mean age ( ± standard deviations) for the cases and controls were 57.55± 10.42 and 61.03± 8.93% respectively. The determinants of poor glycemic control were age (Adjusted odd ratio (AOR)= 0.08; 95% CI= 0.02-0.33), inadequate physical exercise (AOR = 5.05; 95% CI = 1.99-11.98), presence of comorbidities (AOR = 5.50; 95% CI = 2.06-14.66), non-adherence to anti-diabetes medications (AOR= 2.76; 95% CI= 1.19-6.40), persistent proteinuria (AOR=4.95; 95% CI=1.83-13.36) and high-density lipoprotein less than 40 mg/dl (AOR=3.08; 95% CI= 1.30-7.31).

**Conclusions:**

Age less than 65 years, inadequate physical exercise, presence of comorbidities, non-adherence to anti-diabetes medications, persistent proteinuria, and high-density lipoprotein less than 40 mg/dl were the determinants of poor glycemic control. Therefore, targeted educational and behavioral modification programs on adequate exercise and medication adherence should be routinely practiced. Furthermore, early guideline-based screening and treatment of comorbidities and complications is required to effectively manage diabetes mellitus.

## Background

Diabetes mellitus (DM) is a metabolic disorder that is characterized by chronic hyperglycemia due to defects in insulin secretion, insulin action, or both. Type 2 diabetes accounts for more than 90% of all diabetes worldwide (1). The glycemic control of DM patients can be delineated as adequate glycemic control when blood sugar level is near to normal and chances of DM-related complications are low, and poor glycemic control when blood sugar level is too high and chances of diabetes-related complications are high (2). Globally, more than 537 million adults (10.5% of the world’s adult population) live with diabetes. This number is predicted to rise to 643 million adults by 2030 (11.3% of the world’s adult population) due to changes in lifestyle. Of these, more than 50% of diabetes live in low- and middle-income countries (3). Different studies in Sub- Saharan Africa showed that the burden of DM in Sub-Saharan countries is increasing despite low infrastructures for diabetic care (4, 5). Studies done in Ethiopia showed that the prevalence of DM was in the range of 5.1% - 14% (6, 7).

Studies done in different parts of Sub-Saharan Africa revealed that the majority of DM patients had poor glycemic control (8, 9). Similarly, different hospital-based studies done in Ethiopia showed that the burden of poor glycemic control ranged 52% -80% (10–12), and diabetes-related burden is expected to be high.

The morbidity and mortality of DM is due to uncontrolled hyperglycemia related to glycated products (2). Multiple clinical trials showed that intensive blood glucose control correlated with a reduction in complications of DM (13, 14). Early intensive glycemic control of type 2 DM is associated with reduction of diabetes mellitus complications persisting for more than 10 years (15).On the contrary, even short periods of hyperglycemia can increase the risk of diabetes-related complications (16). This highlights the importance of establishing good glycemic control as early as possible.

Since blood glucose level is affected by multiple factors, poor glycemic control is multifactorial and tailspins. Different studies on type 2 DM patients showed patients with long duration of diabetes, lower level of education, higher BMI, dyslipidemia, inadequate physical exercise, poor adherence to medications, and not regularly performing home glucose tests were associated with poor glycemic control (17–19). Another study done in Sub-Saharan countries showed that glycemic control is not affected by education-level, presence of comorbidities, smoking, alcohol intake, type of treatments, measuring blood glucose at home, and body mass index (10).

Cross-sectional studies done in different parts of Ethiopia showed that poor glycemic control is associated with illiteracy, long duration of diabetes illness, poor medication adherence, inadequate follow-up, low level of education, combination therapy, presence of comorbidities and poor utilization of glucometer for self-monitoring (11, 17, 20–22).

There is also a program called the Healthy People 2020 Program, which is aimed at reducing the risk of type 2 diabetes and its complications (23). To achieve the goals of this global program, evidence-based interventions targeting determinants of poor glycemic control are essential.

Though there are cross-sectional studies done in Ethiopia, inconsistencies are seen between the variables that are associated with poor glycemic control. In addition, these cross-sectional studies don’t show the relations between the dependent and independent variables. Most studies also use fasting blood sugar (FBS) level to define poor glycemic control, although hemoglobin A1c (HbA1c) is the preferred method. Moreover, previous studies didn’t encompass modifiable cardiovascular risk factors such as dyslipidemia, obesity, physical exercise, and smoking. Therefore, this study aimed to identify determinants of poor glycemic control including modifiable cardiovascular risk factors, by using HbA1c to define poor glycemic control among type 2 diabetes mellitus patients at the diabetes mellitus clinic of the University of Gondar Compressive Specialized Hospital (UoGCSH), Northwest Ethiopia in 2020. The findings of this study will help improve glycemic control and decrease diabetes mellitus complications.

## Materials and methods

### Study settings and design

A hospital-based unmatched case-control study was conducted from June 5 to September 25, 2020, at the University of Gondar Comprehensive Specialized Hospital diabetes mellitus follow-up clinic. The hospital is located in Gondar town, which is 657 km away from Addis Ababa, the capital city of Ethiopia. The hospital has more than 950 beds and serves as a tertiary referral hospital providing specialized care for nearly 15 million people. The diabetes clinic, which was established in 1985, provides service to more than 5000 diabetes mellitus patients per year. On average, 45 DM patients are seen in the clinic daily from Monday to Friday. Routine clinical and laboratory evaluations, which include fasting blood sugar at each visit; annual retinal examination; serum creatinine and simple urine analysis, and cardiovascular disease screening including electrocardiography and echocardiography when needed, are delivered.

### Population

The source population for this study were all type 2 DM patients who had regular follow-up at the diabetes clinic of UoGCSH and the population of study were all type 2 DM patients who visited the diabetes clinic of UoGCSH during the data collection period. Cases were patients with type 2 DM who had poor glycemic control, and controls were patients with type 2 DM who had good glycemic control.

### Sampling size and sampling procedure

Epi Info software version 7.2 was used to calculate sample size with the parameters of significance = 95%, power = 80%, odd ratio = 4.05. The odd ratio was taken from a study conducted in Public Hospitals of the Central Zone, North Ethiopia (24). The case-to-control ratio was 1:1; the proportion of controls with exposure was 57.8%, and the proportion of cases with exposure was 79.3%. Assuming a non-response rate of 5%, the sample size for controls and cases was 90, which makes the overall sample size 180. A convenience sampling procedure was used to recruit study participants.

### Data collection

Data were collected using a structured questionnaire by one medical doctor and one nurse after orientation was given on how to fill out the questionnaire. Patients were interviewed for socio-demographics, alcohol history, smoking history, adherence to medication, and self-care activity. Physical examinations including measurements of height, weight, and blood pressure were performed. Patient records were reviewed to obtain relevant medical histories like duration of illness, documented complications, and comorbidities. Laboratory results that were done within one year, such as: creatinine, lipid profile (LP), and albuminuria from urine analysis were reviewed from patient records. One ml of venous blood was taken from each patient to determine HbA1c through a Cobas kit by a trained nurse during data collection. Laboratory results were given to patients, and diabetes care was given to all patients based on the HbA1c results.

### Measurements

Blood pressure was measured after the participants stood with arms at the sides, feet positioned close together, and relaxed for 5 minutes. The height was measured to the nearest centimeter. Weight was measured to the nearest kg using a calibrated instrument. Body mass index (BMI) was calculated as weight divided by the square of height (kg/m2). Glomerular filtration rate (GFR) was calculated by the CKD-EPI-Creatinine 2009 equation, which uses serum creatinine, age, and sex of patients. The CKD-EPI equation expressed as a single equation = 141 × min (Scr/κ,1)α × max (Scr/κ, 1)-1.209 × 0.993Age × 1.018 [if female] _ 1.159 [if black], where Scr is serum creatinine, κ is 0.7 for females and 0.9 for males, α is -0.329 for females and -0.411 for males, min indicates the minimum of Scr/κor 1 (25).

### Data quality assurance

The structured questionnaire was developed from different related studies. Data quality was assured by checking the completeness of filled questionnaires and supervision. The questionnaire was pretested on 10% of diabetes mellitus patients of the total sample size. Those patients who were included in the pre-testing were not included in the actual data collection. After the collection of data, a specific marker (HbA1c-PGC) on the chart was used to avoid repetition.

### Study variables

The dependent variable of this study was poor glycemic control whereas the independent variables were socio-demographic characteristics (age, sex, religion, marital status, residency, educational status, economic status, and occupation), lifestyle-related characteristics (exercise, alcohol drinking, and smoking), DM and comorbidity related characteristics (duration of DM, presence of comorbidities, and self-monitoring of blood glucose), anti-DM medication-related characteristics (types of anti-DM medications, duration of medications, and adherence to anti-DM medications) and physical examination (BP and BMI), and laboratory results (serum creatinine, proteinuria, serum high-density lipoprotein (HDL), low-density lipoprotein (LDL), total cholesterol, and triglyceride (TAG).

### Operational definitions

Type 2 DM patients stands for those adults diagnosed by a physician as having Type 2 DM. Good glycemic control is defined as HbA1c less than 8%, where as Poor glycemic control is HbA1c greater than or equal to 8% (2)

Hemoglobin A1c (HbA1c) test is a blood test that shows an average of the blood sugar level over the past 90 days and represents it in percentage. The main advantage of HbA1c is to reflect the cumulative glycemic control of the body in the preceding two to three months and correlates well with the risk of long-term diabetes complications unlike FBS. The drawback of HbA1c is that the result will not be reliable in conditions affecting red blood cell life span, hemoglobin, and anemia (26)

Adherence coincides with patients behavior to stick to professional advice, relationship as part of the process of care, outcome and process targets, taking the medication as prescribed (27). Adherent Patients are those who score 0 based on Morisky Medication Adherence Scale-8 (MMAS-8) and non-adherent Patients are those who score 1–8 based on Morisky Medication Adherence Scale-8 (MMAS-8) (28).

Adequate physical exercise is engagement in moderate-to-vigorous intensity exercise for 150 minutes or more per week for at least 3 days/week, with no more than 2 consecutive days without activity (29). Smoker refers to a person who has smoked at least 100 cigarettes in his or her lifetime (30). A bad alcohol drinking habit is drinking more than a standard drink or those with the habit of binge drinking or who have an alcohol addiction (31).

### Data processing and analysis

The collected data were entered and cleaned with Epidata version 4.6.0.2 and exported to Stata version 14 software packages for data management (extraction, re-coding, and categorization) and statistical analysis (for assessing determinants of poor glycemic control). The data were recoded as Bernoulli random variable and analyzed for its descriptive statistics, and both bivariable and multivariable multilevel analysis were employed. Variables with a p-value <0.20 in the bivariable analysis were eligible for multivariable analysis. In the multivariable analysis, an adjusted odds ratio (AOR) with a 95% confidence interval (CI) was reported and variables with a p-value <0.05 were taken as significant predictors of poor glycemic control.

### Ethical consideration

Ethical approval was obtained from the University of Gondar, College of Medicine and Health Sciences, School of Medicine, Ethical Review Committee with the reference number of SoM1887/02/2020. Study participants were recruited after informed oral consent was obtained. Participants were informed about the objective, benefit, and procedure of the study. HbA1c was determined from serum for free. For those who had poor glycemic control, care was given as per the recommendations of American diabetes association (ADA) guidelines.

## Results

### Socio-demographic characteristics of participants

The study was conducted on 180 type 2 diabetes mellitus patients, 90 cases, and 90 controls, of which 53.3% and 54.4% were females, respectively. The mean ± SD age of the respondents was 57.55 ± 10.42 and 61.03± 8.93% of cases and controls, respectively. The majority of the study participants (46.7% of cases; 47.8% of controls) were in the age group “50–64 years”. Three-fourths of cases and two-thirds of controls were married. Thirty-seven percent of cases and 44.4% of controls had no formal education. More than 90% of participants for both cases and controls live in urban areas (**Table 1**).

**Table 1:** Socio-demographic characteristics of type 2 diabetes mellitus patients at DM clinic of UoGCSH, Northwest Ethiopia, 2020 (Total number (N)=180) .

Variables	Glycemic control		Total N (%)
	Case N (%)	Control N (%)	
Age category			
35-49	22 (24.4)	7 (7.8)	29 (16.1)
50-64	42 (46.7)	43 (47.8)	85 (47.2)
65-80	26 (28.9)	40 (44.4)	66 (36.7)
Gender			
Male	42 (46.7)	41 (45.6)	83 (46.1)
Female	48 (53.3)	49 (54.4)	97 (53.9)
Place residence			
Urban	84 (93.3)	81 (90)	165 (91.7)
Rural	6 (6.7)	9 (10)	15 (8.3)
Marital status			
Married	68 (75.6)	62 (68.9)	130 (72.2)
Single/widowed/divorced	22 (24.4)	28 (31.1)	50 (27.8)
Educational status			
No formal education	33 (36.7)	40 (44.4)	73 (40.6)
Primary school	17 (18.9)	7 (7.8)	24 (13.3)
Secondary school	18 (20)	18 (20)	36 (20)
College and above	22 (24.4)	25 (27.8)	47 (26.1)
Occupation			
Farmer	19 (21.1)	12 (13.3)	31 (17.2)
Merchant	7 (7.8)	17 (18.9)	24 (13.3)
Housewife and Pensioner	33 (36.7)	38 (42.2)	71 (39.4)
Employee*	31 (34.4)	23 (25.6)	54 (30)
Monthly income, ETB			
≤ 2000	37 (41.1)	35 (38.9)	72 (40)
> 2000	53 (58.9)	55 (61.1)	108 (60)

*Government and nongovernmental employee, ETB= Ethiopian birr; at time of data collection 1$= 35 ETB.

### Diabetes self-care activities and behavioral characteristics of participants

This study showed that one hundred seventy-six (97.8%) of the study participants were nonsmokers, whereas 89% of cases and 86.7% of controls had no bad drinking habits. More than one-third (33.3%) of the cases and 81% of the controls have been involved in regular adequate physical exercise. Less than a quarter of participants in both cases (21%) and controls (24%) check their blood glucose on regular basis at home (**Table 2**).

**Table 2:** Behavioral characteristics and diabetes self-care activities in type 2 diabetes mellitus patients at DM clinic of UoGCSH, Northwest Ethiopia, 2020 (N=180).

Variables	Glycemic control		Total N
	Cases N (%)	Controls N (%)	
Smoking status			
Smoker	1 (1.1)	3 (3.3)	4 (2.2)
Non-smoker	89 (98.9)	87 (96.7)	176 (97.8)
Alcohol habit, drinks/d			
None or <2	10 (11.1)	12 (13.3)	22 (12.2)
More than 2	80 (88.9)	78 (86.7)	158 (87.8)
Physical exercise			
Adequate	39 (43.3)	70 (77.8)	109 (60.6)
In adequate	51 (56.7)	20 (22.2)	71 (39.4)
Self-monitoring blood glucose			
Yes	22 (24.4)	19 (21.1)	41 (22.8)
No	68 (75.6)	71 (78.9)	139 (77.2)

### Selected clinical characteristics of participants

**Figure 1:**
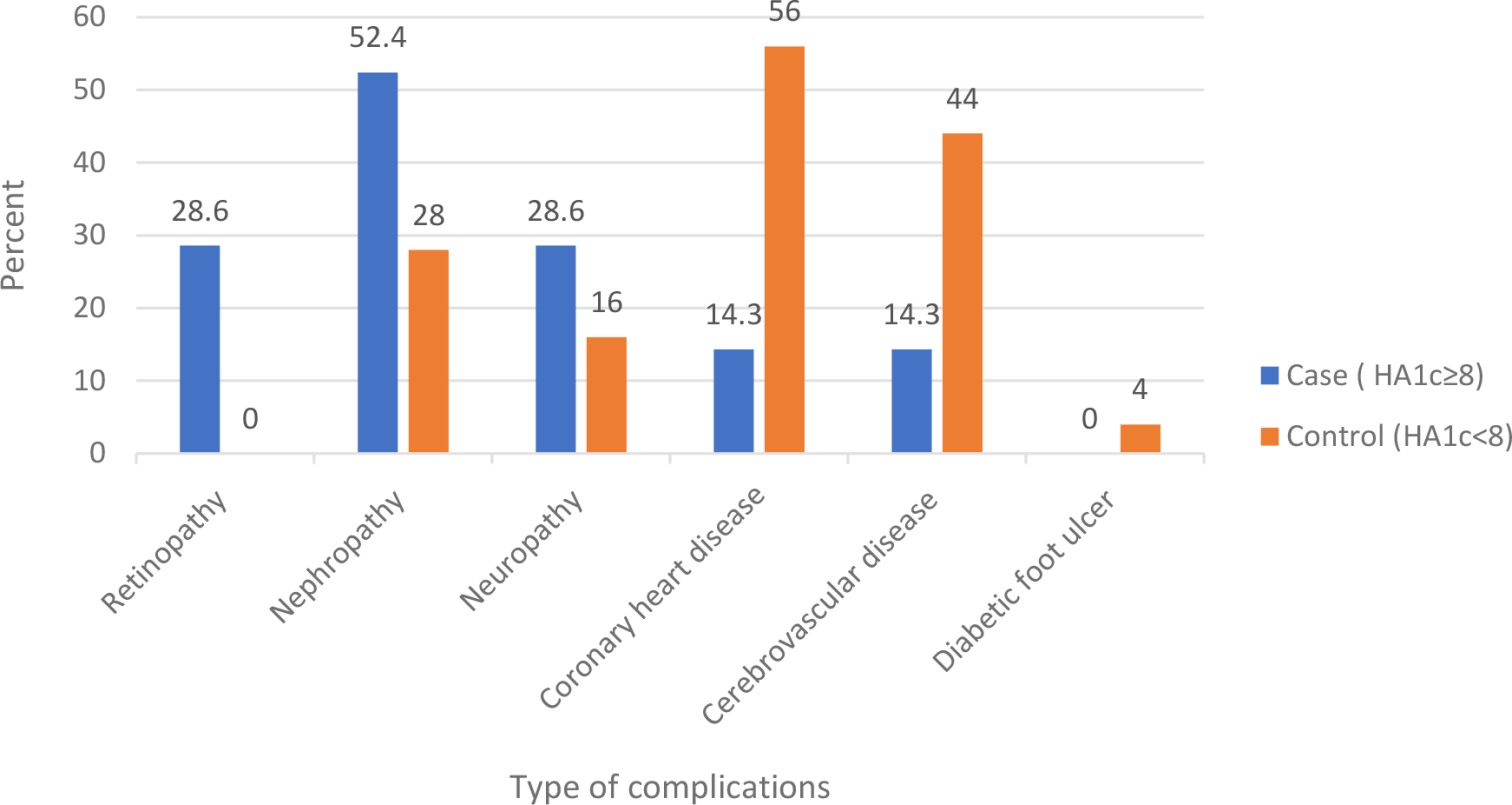
Complications among type 2 diabetes mellitus patients at DM clinic of UoGCSH, Northwest Ethiopia, 2020.

**Figure 2:**
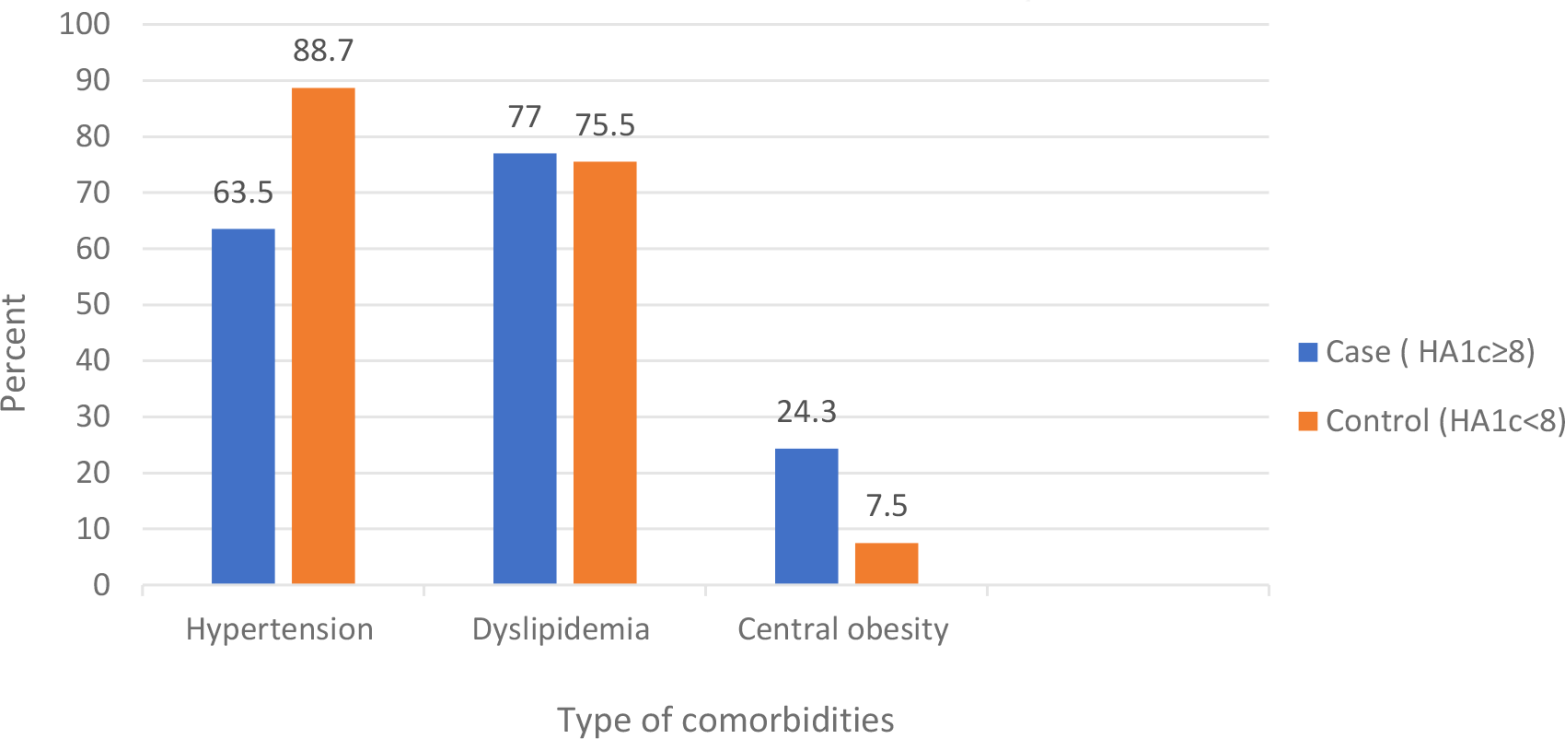
Comorbidities among type 2 diabetes mellitus patients at DM clinic of UOGCSH, Northwest Ethiopia, 2020.

The duration of diabetes was greater than 7 years in 61.1% of the cases and 71.1% of the controls. Less than a quarter of cases (23.3%) and 27.8% of controls had DM-associated complication. 52.4% of cases, and 28% of control had nephropathy. Fifty six percent of controls, and 14.3% of cases had coronary heart disease. Forty four percent of controls and 14.3% of cases had cerebrovascular disease. Eighty-two percent of cases and 58.9% of controls had comorbidities, of which 88.7% of controls had hypertension, and 77% of cases had dyslipidemia. Less than half of the participants (43.3% of cases and 45.6%of controls) had normal body mass index **Table 3** and **Figures 1**, **2**.

**Table 3:** Clinical characteristics of type 2 diabetes mellitus patients at DM clinic of UoGCSH, Northwest Ethiopia, 2020 (N=180).

Variables	Glycemic control		Total N
	Cases N (%)	Controls N (%)	
Duration of diabetes in years			
≤7years	55 (61.1)	64 (71.1)	119 (66.1)
>7 years	35 (38.9)	26 (28.9)	61 (33.9)
Complications			
Yes	21 (23.3)	25 (27.8)	46 (25.6)
No	69 (76.6)	65 (72.2)	134 (74.4)
Comorbidity			
yes	74(82.2)	53 (58.9)	127 (70.6)
No	16 (17.8)	37 (41.1)	53 (29.4)
Body mass index in kg/m^2^			
Normal (18.5-24.9 kg/m^2^)	39 (43.3)	41 (45.6)	80 (44.4)
Overweight (25-29.9 kg/m^2^)	35 (38.9)	36 (40)	71 (39.4)
Obese (≥30 kg/m^2^)	16 (17.8)	13 (14.4)	29 (16.1)
Current SBP in mm Hg			
Normal (<140 mmHg)	45 (50)	35 (38.9)	80 (44.4)
Elevated (≥140 mmHg)	45 (50)	55 (61.1)	100 (55.6)
Current DBP in mm Hg			
Normal (<90 mmHg)	46 (51.1)	39 (43.3)	85 (47.2)
Stage 1 (≥90 mmHg)	44 (48.9)	51 (56.7)	95 (52.8)

SBP, systolic blood pressure; DBP, diastolic blood pressure.

### Medication-related characteristics of participants

**Figure 3:**
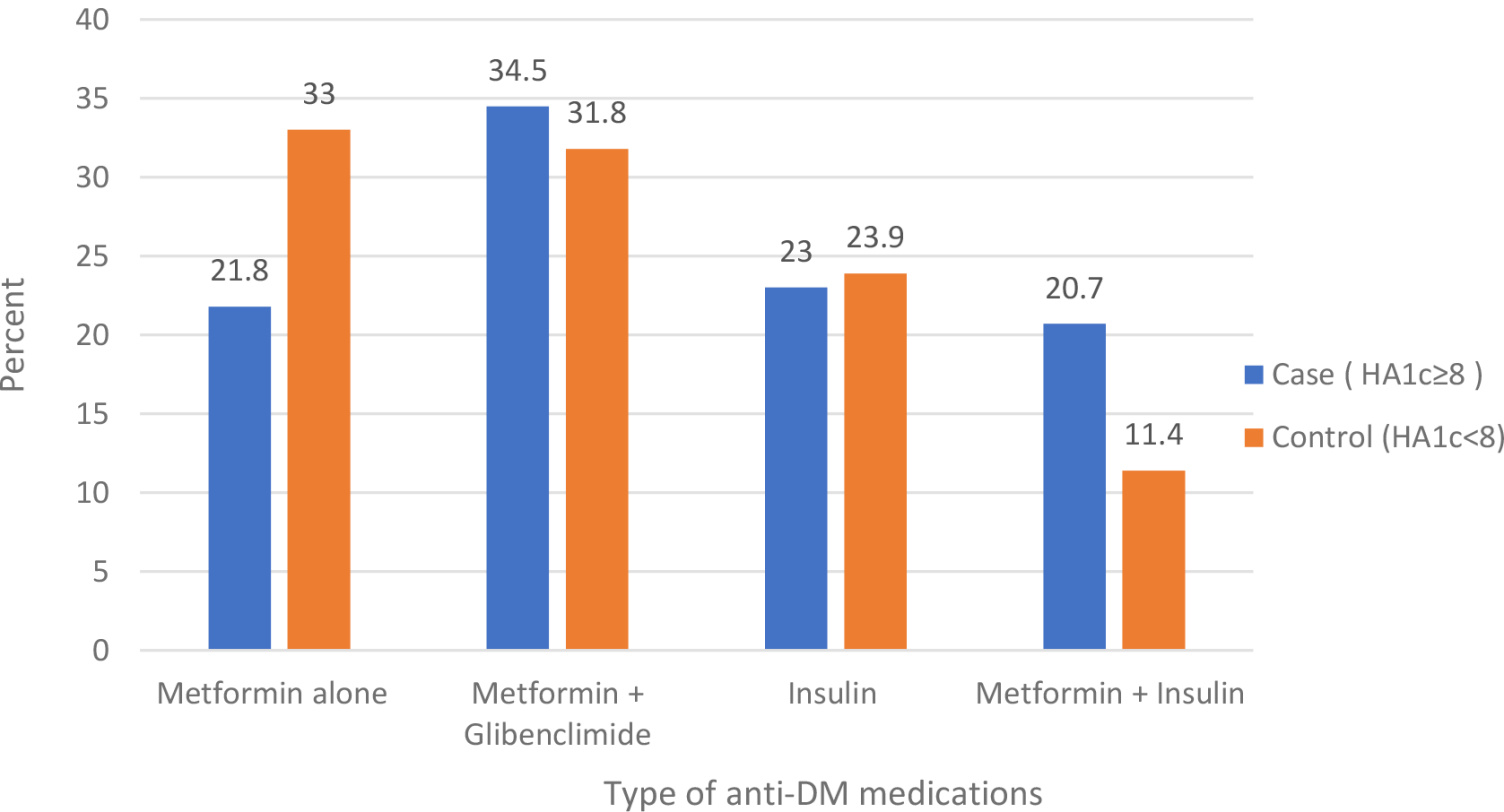
Type of anti-DM medications among type 2 diabetes mellitus patients at the DM clinic of UOGCSH, Northwest Ethiopia, 2020.

In this study, almost all (97.2%) of the participants were on medication; of these, one-third of participants were on dual oral anti-diabetes medication in both cases and controls. Less than a quarter of participants in cases (23%) and controls (24%) were on insulin. Among those on medication, more than half of cases (54%) and 9.1% of controls were not adherent to their medications (**Table 4**; **Figure 3**)

**Table 4:** Medication-related characteristics of type 2 diabetes mellitus patients at DM clinic of UoGCSH, Northwest Ethiopia, 2020 (N=180).

Variables	Glycemic control		Total N
	Cases N (%)	Controls N (%)	
Medication status			
On medication	87 (96.7)	88 (97.8)	175 (97.2)
Not on medication	3 (3.3)	2 (2.2)	5 (2.8)
Type of medication			
Metformin alone	19 (21.8)	29 (33)	48 (27.4)
Metformin + Glibenclimide	30 (34.5)	28 (31.8)	58 (33.1)
Insulin	20 (23)	21 (23.9)	41 (23.4)
Metformin + Insulin	18 (20.7)	10 (11.4)	28 (16)
Medication adherence status			
Non-adherent	47 (54)	21 (23.9)	68 (38.9)
Adherent	40 (46)	67 (76.1)	107 (61.1)

### Selected biochemical parameters of participants

For all participants, laboratory profiles such as fasting blood sugar, lipid profile, serum creatinine, and urine analysis were determined. The majority (82.2%) of participants from cases and more than half (54.5%) of participants from controls had fasting blood glucose level of more than 140mg/dl. Fifty percent of cases and 14.4% of controls had persistent proteinuria. The mean total cholesterol was 40.22 ± 10.22 in cases and 38.8 ± 9.22 in controls. More than half (58.9% in cases and 53.3% in controls) of the participants had LDL higher than 100mg/dl (**Table 5**).

**Table 5:** Biochemical parameters of type 2 diabetes mellitus at DM clinic of UOGCSH, Northwest Ethiopia, 2020 (N=180).

Variables	Glycemic control		Total N
	Cases N (%)	Controls N (%)	
Fasting blood sugar			
<140 mg/dl	16 (17.8)	41 (45.6)	57 (31.7)
≥ 140 mg/dl	74 (82.2)	49 (54.4)	123 (68.3)
Serum creatinine			
≤1mg/dl	77 (85.6)	73 (81.1)	150 (83.3)
>1mg/dl	13(14.4)	17 (18.9)	30 (16.7)
Glomerular filtration rate			
Stage 1	49 (54.4)	47 (52.2)	96 ()53.3
Stage 2	30 (33.3)	34 (37.8)	64 (35.6)
Stage 3	11 (12.2)	9 (1o)	20 (11.1)
Proteinuria			
Yes	36 (40)	13 (14.4)	49 (27.2)
No	54 (60)	77 (85.6)	131 (72.8)
LDL			
< 100mg/dl	37 (41.1)	42 (46.7)	79 (43.9)
≥100mg/dl	53 (58.9)	48 (53.3)	101 (56.1)
HDL			
≥ 40mg/dl	41 (45.6)	51 (56.7)	92 (51.1)
< 40mg/dl	49 (54.4)	39 (43.3)	88 (48.9)
Total cholesterol			
< 200 mg/dL	58 (64.4)	66 (73.3)	124 (68.9)
≥200 mg/dl	32 (35.6)	24 (26.7)	56 (31.1)
Triglyceride			
<150mg/dl	38 (42.2)	45 (50)	83 (46.1)
≥150mg/dl	52 (57.8)	45 (50)	97 (53.9)

### Predictors of poor glycemic control

In bivariate logistic regression; age, educational level, physical exercise, duration since diagnosis of diabetes, presence of comorbidities, current systolic blood pressure (SBP), types of anti-diabetes mellitus medication, adherence to anti-diabetes mellitus medication, persistent proteinuria, and HDL level were found to be associated with poor glycemic control and entered into the multivariate logistic analysis. In multivariable analysis; age less than 65, inadequate physical exercise, presence of comorbidities, non-adherence to anti-diabetes mellitus medication, persistent proteinuria, and low HDL level were found to be significantly associated with poor blood glycemic control as depicted in **Table 6**.

**Table 6:** Predictors of poor glycemic control among type 2 diabetes mellitus patients at DM clinic of UoGCSH, Northwest Ethiopia, 2020 (N=180).

Variables	Glycemic control		COR (95% CI)	AOR (95% CI)
	Cases N (%)	Controls N (%)		
Age category				
35-49	22 (24.4)	7 (7.8)	1	1
50-64	42 (46.7)	43 (47.8)	0.31 (0.12-0.80)	0.22 (0.06-0.75) *
65-80	26 (28.9)	40 (44.4)		0.08 (0.02-0.33) *
Educational status				
No formal education	33 (36.7)	40 (44.4)	0.93 (0.44-1.95)	1.97 (0.29-2.57)
Primary school	17 (18.9)	7 (7.8)	1.25 (0.96-7.88)	1.31 (0.32-4.86)
Secondary school	18 (20)	18 (20)	0.98 (0.47-2.70)	1.17 (0.31-3.15)
College and above	22 (24.4)	25 (27.8)	1	1
Physical exercise				
Adequate	39 (43.3)	70 (77.8)	1	1
Not adequate	51 (56.7)	20 (22.2)	4.89 (2.39-8.75)	5.05 (1.99—11.98) *
Duration of DM				
≤ 7 years	55 (61.1)	64 (71.1)	1	1
> 7 years	35 (38.9)	26 (28.9)	1.56 (0.84- 2.91)	1.52 (0.61-3.74)
Comorbidity				
Yes	74(82.2)	53 (58.9)	3.22 (1.63- 6.40)	5.50 (2.06-14.66) *
No	16 (17.8)	37 (41.1)	1	1
Current SBP, mm Hg				
Normal (<140 mm Hg)	45 (50)	35 (38.9)	1	1
Elevated (≥140 mm Hg)	45 (50)	55 (61.1)	0.63 (0.35-1.15)	0.49 (0.21-1.16)
Type of medication				
Metformin alone	19 (21.8)	29 (33)	1	1
Metformin+ Glibenclimide	30 (34.5)	28 (31.8)	1.42 (0.75-3.54)	1.65 (0.51-3.93)
Insulin	20 (23)	21 (23.9)	0.86 (0.62-3.37)	1.04 (0.27-2.66)
Metformin + insulin	18 (20.7)	10 (11.4)	1.66 (1.04-7.21)	1.80 (0.44-6.17)
Medication adherence status				
Adherent	47 (54)	21 (23.9)	1	1
Non-adherent	40 (46)	67 (76.1)	3.87 (2.04-7.35)	2.76 (1.19-6.40) *
Persistent proteinuria				
Yes	36 (40)	13 (14.4)	3.95 (1.91-8.13)	4.95(1.83-13.36) *
No	54 (60)	77 (85.6)	1	1
HDL				
≥ 40mg/dl	41 (45.6)	51 (56.7)	1	1
< 40mg/dl	49 (54.4)	39 (43.3)	1.5 6 (0.87-2.81)	3.08 (1.30-7.31) *

*Determinant of poor glycemic control; OR, odd ratio.

Participants whose ages were between 50-64 years were less likely to have poor glycemic control than those aged 35-49 (AOR=0.22; 95% CI=0.06-0.75). Similarly, participants 65 years and above had less chance of exhibiting poor glycemic control than those aged 35-49 (AOR= 0.08; 95% CI= 0.02-0.33).

Respondents who didn’t engage in adequate physical exercise had a high chance of having poor glycemic control compared to those engaged in physical exercise (AOR = 5.05; 95% CI = 1.99—11.98). Participants who had comorbidities were more likely to have poor glycemic control compared to those who had no comorbidities (AOR = 5.50; 95% CI = 2.06-14.66)

Respondents who were not adherent to their medication were more likely to have poor glycemic control than those who were adherent (AOR= 2.76; 95% CI= 1.19-6.40). Participants whose HDL level was less than 40 mg/dl had poor glycemic control than those who had values greater than 40 mg/dl (AOR=3.08; 95%CI= 1.30-7.31). Respondents who had persistent proteinuria on urine analysis had high chance of being characterized as having poor glycemic control than those who had no persistent proteinuria on urine analysis (AOR=4.95; 95%CI=1.83-13.36).

## Discussion

The main target in the management of DM is maintaining good glycemic control, which is very important for controlling and preventing diabetes mellitus complications (32). Glucose measurement is the main tool for assessing glycemic control. The American Diabetes Association recommends an HbA1c level below 7% for those who have no diabetes-related complications and young age groups, and less than 8% for those who have complications or comorbidities that threaten life expectancy as a target for optimal blood glucose control (2). Knowing the factors associated with poor glycemic control is important for clinical intervention and better treatment outcomes in diabetes mellitus patients.

This case-control study assessed factors that predict poor glycemic control among type 2 diabetes mellitus patients at University of Gondar Comprehensive Specialized Hospital Diabetes Clinic. In this study, participants who were younger than 65 years old, had inadequate physical exercise, with comorbidities, non-adherent to anti-diabetes mellitus medications, persistent proteinuria, and high-density lipoprotein level less than 40 mg/dl were found significantly associated with poor blood glycemic control.

Participants who were in the age range of 50-64 years were less likely to have poor glycemic control than those at the age of 35-49 years. Similarly, those whose age is in the range of 65-80 years have less chance of having poor glycemic control. On the same line of this study, studies done in South Sahara (9), Uganda (33), Malaysia (34), Iran (35), Germany (36), Singapore (37, 38), and United States of America (39, 40) showed that older patients had better glycemic control than the younger age group. This might be due to weak self-management behavior like doing regular physical exercise, glucose testing, medication adherence, and diet modification among young patients as compared to older patients (41). Educating the younger population with diabetes on significance of proper self-care habits should be emphasized since this group of patients may have longer life expectancy, and it is important to prevent complications associated with diabetes. In contrary to this study, cross-sectional studies in central Ethiopia (42), India (43), and Nigeria (44) showed that poor glycemic control in adults with type-2 diabetes was higher with age greater than 65 years. The difference might be due to differences in method, sample size, and country of origin with all the cultural and social differences that may imply.

Respondents with inadequate physical exercise were more likely to have poor glycemic control compared to those with adequate regular physical exercise. The results of this study are consistent with previous studies conducted in Ethiopia (6, 24, 45–47), Libya (48), and Jordan (19). The reason might be because working muscles consume higher glucose than muscles at rest, and physical activity increases blood flow to these muscles and eventually increases the number of insulin receptors, which finally results in increased insulin sensitivity (49). Another possible reason is that exercise decreases central obesity, bad cholesterol, and plasma norepinephrine which will in turn decrease serum glucose level. Effective education on advantages of exercise programs and adherence to exercise should be done during follow-up. However, the findings in this study are different from the cross-sectional study conducted in Ethiopia (50). The difference could be due to a divergence in exercise measurements, sample size, and design.

Patients who have one or more comorbidities have higher odds of having poor glycemic control, a result similar to what is reported in previous studies in Ethiopia (10, 45, 51). Similarly, a study conducted in Iran (52), Colombia (53), and North America (54) showed that type 2 diabetes mellitus patients who had comorbidities had a higher chance of poor glycemic control than those who didn’t have complications. The reason for respondents with comorbidities having poor glycemic control might be due to poor adherence to medication because of additional medication pill burden. The other possible reason may be patients with comorbidities will have low motivation for self-management of diabetes mellitus, which is a corner stone for glycemic control. Additionally, some comorbidities like abdominal obesity, may increase insulin resistance and decrease peripheral glucose uptake (53). To improve glycemic control: comorbidities should be addressed and managed accordingly; barriers to adherence to medication should be addressed, and those with comorbidities should be put on close follow-up to support self-management practices. On the contrary, studies conducted in southwest Ethiopia (21), eastern Sudan (55), and Thailand (56) showed that comorbidities were not associated with poor glycemic control. The difference might be due to different study methodology and clinical approach to comorbidities treatment at the site.

Non-adherence to diabetes medication was found significantly associated with poor glycemic control, which was in line with cross-sectional studies conducted in different parts of Ethiopia (21, 57, 58), Libya (48), and Dar es Salaam (33). The reason could be that non-adherence to anti-diabetes medication may expose the patient to high blood glucose levels due to increasing glucose production from the liver, decreasing insulin secretion from the beta-cells, or decreasing glucose uptake by skeletal muscles (59). To achieve good glycemic control, barriers to medication adherence should be addressed, and effective educational and behavioral intervention programs on adherence to medications need to be conducted (60). On the contrary, in a study done at Tikur-Anbessa specialized hospital (61) medication adherence was not associated with glycemic control. The possible reason might be due to different adherence assessment tools, sample size, and design. The other difference is that FBS was used for poor glycemic definition in the previous study, while HbA1C was used in this study

Persistent proteinuria in urine analysis was associated with poor glycemic control. Participants who had persistent proteinuria were more likely to have poorly controlled blood glucose level than those without persistent proteinuria. The most common cause of persistent proteinuria in diabetes mellitus is diabetes nephropathy, which is one complication of poor glycemic control. Similarly, studies conducted in Ethiopia (45, 62), Egypt (63), and Kenya (64) also showed that patients who had diabetes nephropathy had higher chances of poor glycemic control than those with no diabetic nephropathy. Screening for microvascular complications like nephropathy and guideline-directed treatment should be carried out during follow-up.

Respondents whose high-density lipoprotein level is less than 40 mg/dl have higher chances of having poorly controlled blood glucose than those with HDL levels greater than 40 mg/dl. Similar to this study, studies done in Sudan (65), India (43), and Saudi Arabia (66) showed that respondents with HDL levels less than 40 mg/dl had poor glycemic control than those who had HDL levels greater than 40 mg/dl. The possible reason for this might be impaired liver apolipoprotein production, which in turn regulates enzymatic activity of lipoprotein lipase (LPL) and cholesterol ester transport protein . Therefore, yearly screening for dyslipidemia and guideline-based treatment should be adopted.

## Strengths and limitations of the study

### The strength of the study

Since this study was case-control, its strength in identifying determinants of poor glycemic control is better than cross-sectional studies done in Ethiopia previously. In this study, HbA1c was used to evaluate glycemic control, which is better than FBS used in most previous studies in Ethiopia.

### Limitations of the study

As some parts of the questionnaire depended on the memory of respondents, it may have resulted in recall bias. It was done only among type 2 diabetes mellitus patients who were on follow-up at a governmental hospital, which may not be representative of the overall type 2 diabetes population

## Conclusions

Age younger than 65 years, inadequate physical exercise, the presence of comorbidities, non-adherence to diabetes medications, persistent proteinuria, and HDL level less than 40mg/dl were the independent predictors of poor glycemic control. Therefore, targeted educational and behavioral modification programs on regular exercise, and medication adherence should be routinely practiced. Additionally, early guideline-based screening and treatment of comorbidities and complications would be required to effectively manage diabetes mellitus.

## Data availability statement

The raw data supporting the conclusions of this article will be made available by the authors, without undue reservation.

## Ethics statement

The studies involving human participants were reviewed and approved by University of Gondar, College of Medicine and Health Sciences School of Medicine Ethical Review Committee. The patients/participants provided their written informed consent to participate in this study.

## Author contributions

GL is involved in conceiving the idea, study design, data analysis and interpretation, and managing the overall progress of the study and manuscript preparation. GA, TY and SA are equally involved in study design, and follow-up of study. Both TA and YT equally contributed to data analysis and interpretation, and in revising the manuscript. All authors contributed to the article and approved the submitted version.
