# Corrigendum: Determinants of poor glycemic control among type 2 diabetes mellitus patients at University of Gondar Comprehensive Specialized Hospital, Northwest Ethiopia: Unmatched case-control study

**DOI:** 10.3389/fendo.2023.1220605

**Published:** 2023-06-20

**Authors:** Gebrehiwot Lema Legese, Getahun Asres, Shitaye Alemu, Tesfaye Yesuf, Yeabsira Aklilu Tesfaye, Tsegaw Amare

**Affiliations:** ^1^ Department of Internal Medicine, School of Medicine, College of Medicine and Health Sciences, University of Gondar, Gondar, Ethiopia; ^2^ Department of Health Systems and Policy, Institute of Public Health, College of Medicine and Health Sciences, University of Gondar, Gondar, Ethiopia

**Keywords:** determinants, diabetes mellitus, glycemic control, HbA1C, Ethiopia

In the published article, there was a mistake in the first paragraph of abstract. Instead of “Poor glycemic control is one of the most determinant factors for type 2 diabetes-related morbidity and mortality. The proportion of type 2 diabetes mellitus patients with poor glycemic control remains high. Yet evidence on factors contributing to poor glycemic control remains scarce. The aim of this study is to identify determinants of poor glycemic control among type 2 diabetes mellitus patients at a diabetes mellitus clinic in University of Gondar Comprehensive Specialized Hospital, Northwest Ethiopia Determinants of Poor Glycemic Control among Type 2 Diabetes mellitus Patients at University of Gondar Comprehensive Specialized Hospital, Northwest Ethiopia: Unmatched Case-Control Study”, it should be “Poor glycemic control is one of the most determinant factors for type 2 diabetes-related morbidity and mortality. The proportion of type 2 diabetes mellitus with poor glycemic control remains high. Yet evidences on factors contributing to poor glycemic control remain scarce. The aim of this study is to identify determinants of poor glycemic control among type 2 diabetes mellitus patients at a diabetes mellitus clinic in University of Gondar Comprehensive Specialized Hospital, Northwest Ethiopia”.

The authors apologize for this error and state that this does not change the scientific conclusions of the article in any way. The original article has been updated.

